# No prominent role for complement C1-esterase inhibitor in Marfan syndrome mice

**DOI:** 10.1530/VB-22-0016

**Published:** 2022-10-24

**Authors:** Stijntje Hibender, Siyu Li, Alex V Postma, Myrthe E Hoogeland, Denise Klaver, Richard B Pouw, Hans W Niessen, Antoine HG Driessen, David R Koolbergen, Carlie JM de Vries, Marieke JH Baars, Arjan C Houweling, Paul A Krijnen, Vivian de Waard

**Affiliations:** 1 Amsterdam UMC Location University of Amsterdam, Department of Medical Biochemistry, Meibergdreef, Amsterdam, The Netherlands; 2 Amsterdam Cardiovascular Sciences, Atherosclerosis & Ischemic Syndromes, Amsterdam, The Netherlands; 3 Amsterdam UMC Location University of Amsterdam, Department of Medical Biology, Meibergdreef, Amsterdam, The Netherlands; 4 Amsterdam UMC Location University of Amsterdam, Department of Human Genetics, Meibergdreef, Amsterdam, The Netherlands; 5 Sanquin Research, Department of Immunopathology, Plesmanlaan, Amsterdam, The Netherlands; 6 Amsterdam UMC Location University of Amsterdam, Landsteiner Laboratory, Meibergdreef, Amsterdam, The Netherlands; 7 Amsterdam UMC Location University of Amsterdam, Department of Pathology, Meibergdreef, Amsterdam, The Netherlands; 8 Amsterdam UMC Location University of Amsterdam, Heart Center, Department of Cardiothoracic Surgery, Meibergdreef, Amsterdam, The Netherlands

**Keywords:** aortic aneurysm, complement inhibition, Inflammation, Marfan syndrome, whole genome sequencing

## Abstract

Marfan syndrome (MFS) is a connective tissue disorder causing aortic aneurysm formation. Currently, only prophylactic aortic surgery and blood pressure-lowering drugs are available to reduce the risk of aortic rupture. Upon whole genome sequencing of a Marfan family, we identified a complement gene C1R variant (p.Ser152Leu), which is associated with severe aortic patients. Therefore, we assessed the role of complement activation in MFS aortic tissue. Expression of various complement genes and proteins was detected in human and murine MFS aneurysm tissue, which prompted us to study complement inhibition in MFS mice. Treatment of the *Fbn1*^C1041G/+^ MFS mice with human plasma-derived C1-esterase inhibitor Cetor® resulted in reduced complement deposition, decreased macrophage influx in the aorta, and lower circulating TNFα levels. However, in line with previous anti-inflammatory treatments, complement inhibition did not change the aortic dilatation rate in this MFS mouse model. Thus, while complement factors/component 3 activation were detected in human/murine MFS aorta, Cetor® had no effect on aortic dilatation in MFS mice, indicating that complement inhibition is not a suitable treatment strategy in MFS.

## Introduction

Marfan syndrome (MFS) is an autosomal dominant connective tissue disorder, which is caused by mutations in the fibrillin-1 (*FBN-1*) gene (1). Pathogenic variants cause the hallmark features of the syndrome, including aortic aneurysm formation. Aortic dilatation often results in aortic dissection/rupture, with mostly fatal consequences (2). Current treatment consists of blood pressure-lowering drugs, such as β-blockers and angiotensin-II receptor blockers, which are ineffective to avoid prophylactic aortic surgery (3). Thus, new pharmacological treatment options are desirable to improve aortic health.

We have previously observed inflammation in the aorta of MFS patients (4). The complement pathway contributes to the innate immune system, by specific targeting of damaged cells. It involves the activation of component 3 (C3), which can be achieved via three routes (5). In the classical pathway, C1 activation takes place by antigen–antibody complexes or apoptotic cells. The lectin and alternative pathways become activated by carbohydrate constituents on the surface of pathogens or damaged cells. Moreover, external factors, such as proteases from the coagulation cascade, can trigger the complement system. Next to opsonization, leukocyte activation ultimately results in terminal complement pathway activation, whereby damaged cells are lysed by incorporation of the C5b-9 membrane attack complex into the cell membrane. The lysis of damaged cells may be a relevant complement activation pathway in MFS, as smooth muscle cell (SMC) death is observed in the MFS aorta (6, 7).

Complement activation plays a role in cardiovascular diseases (5, 8), such as complement activation proteins C3d/C4d in the infarcted myocardium (9). Moreover, it has been observed in thoracic aortic aneurysm (TAA) and abdominal aortic aneurysm (AAA) patient material (10, 11, 12, 13) and in murine TAA and AAA models (14, 15, 16). In murine AAA models, the alternative pathway plays a critical role (14), where the lectin pathway precedes the alternative pathway (15). Less SMC-actin (Acta2) in the murine aorta suggests SMC-loss upon lectin pathway activation (13). In a TAA model, the C3a–C3aR axis induced aortic dissection via enhanced matrix metalloproteinase-2 expression (16). In humans, C5a is increased in the blood of AAA patients and has prognostic potential for aneurysm growth (17). In addition, mutations in two proteolytic subunits of the complement system C1-complex, C1r and C1s, cause periodontal Ehlers–Danlos syndrome. Patients with this disorder sometimes develop cerebral aneurysms, TAA or AAA and dissections (18). Furthermore, genetic variants in *C1R* are associated with TAA formation in bicuspid aortic valve (BAV) patients (19). C1r and C1s bind to C1q, which has a collagen-like structure. Perhaps some of these mutations may involve enhanced binding and cleavage of collagens in connective tissues, rather than binding to C1q (18). Collectively, these data imply that there may be a role for complement activation in aneurysm progression in MFS.

The complement system is tightly regulated by inhibitors to prevent spontaneous activation. The imbalance may result in immune dysregulation and excessive tissue damage (8, 20). C1-esterase inhibitor (C1-INH; purified from the blood, and it is known as Cetor®) is the main protease inhibitor to maintain this balance (21). This inhibitor belongs to the serpin superfamily and is produced by hepatocytes, monocytes, macrophages, fibroblasts, and endothelial cells, mainly upon stimulation with interferon-γ (21). While C1-INH prevents activation of the classical pathway, it also blocks proteases in the lectin pathway, thereby preventing the proteolytic cleavage of both complement components C2 and C4 and thus ultimately C3, which is essential to activate the alternative complement cascade (5, 21, 22). C1-INH-based anti-inflammatory therapy has been used in some diseases already, such as sepsis and acute myocardial infarction (23).

We previously demonstrated that treatment with polyphenol resveratrol, which improved endothelial cell function, reduced aortic aneurysm formation in MFS mice (7). Since C1-INH Cetor® could also preserve endothelial cell function in an atherosclerotic vein-graft model in mice (24), we anticipated its beneficial effect in MFS.

While C1-INH Cetor® is mainly known for its function as a complement inhibitor, this inhibitor also blocks proteases of the fibrinolytic, clotting, and kinin-kallikrein pathways (8, 25), and therefore is used in hereditary angioedema patients who have low C1-INH levels. In the kinin-kallikrein pathway, C1-INH is an important physiological inhibitor of plasma kallikrein (26), which is known to cleave pro-renin to generate active renin, activating the renin–angiotensin–aldosterone system. C1-INH Cetor® may thus potentially reduce angiotensin-II generation. Since angiotensin-II receptor 1 (AT1R) antagonism reduces aneurysm formation and aortic events in MFS patients (27, 28, 29) and mice (30), a beneficial effect of C1-INH Cetor® could be expected for just this reason.

We hypothesize that administration of C1-INH Cetor® to MFS mice will result in decreased complement deposition and inflammation, and thereby reduced vascular damage and aortic aneurysm growth.

## Materials and methods

### Whole genome sequencing

Whole genome sequencing (WGS) was performed with permission of the local Amsterdam UMC Ethical Board (reference: W20_019#20.044), carried out in accordance with the Declaration of Helsinki, upon informed consent in six family members with a pathogenic variant in *FBN1* (*FBN1*: c.T937G; p.C313G) causing MFS (31). Three family members (2 female and 1 male) with a severe vascular phenotype (Patient 2: Type A aortic dissection age 56, followed by aortic root replacement (AoRR), 3 years later type B aortic dissection; Patient 3: Aortic root growth of 5–9 mm per year over 4 years, requiring AoRR at age 64; Patient 6: AoRR age 47, and mitral valve prolapse) were compared to 3 family members (2 female and 1 male) with a mild vascular phenotype (Patients 1, 4, and 5; no significant aortic root dilatation (36–41 mm), ages 49–71 years old). TruSeq DNA Sample prep, input 250 ng, and Illumina’s HiSeq X Ten sequencing platform were used according to the manufacturer’s instructions to perform WGS.

Illumina data were processed with the in-house developed pipeline with settings validated for clinical genetics (Version 1.12 https://github.com/hartwigmedical/pipeline/releases/tag/v1.12), covering >95% exome targets (20×/sample). Variant prioritization by Cartagenia Bench Lab NGS (Agilent Technologies) is based on location (exonic and splice-site region variants), population allele frequency, and anticipated inheritance pattern. Further interpretation is based on predicted functional impact and the literature.

### Animal experiments

*Fbn1*^C1041G/+^ male mice were used for the C1-INH (Cetor®) study (*n*  = 8/group) at 2 months old, together with age-matched WT male littermates from a heterozygous breeding colony on C57Bl6-background. Cetor® is a human C1-esterase inhibitor preparation isolated from plasma by Sanquin Plasma Products (Amsterdam, The Netherlands). As such, it has been used as a treatment in biomedical research settings and in hereditary angioedema patients. This human C1-INH was marketed by Shire under the name of Cinryze and as Cetor by Sanquin.

Cetor® was administered via i.v. tail injection 1×/week (15 U in 150 µL water). The mice were euthanized at 4 months old by an overdose of 166 mg/kg ketamine and 24 mg/kg xylazine, perfusion-fixed in 4% paraformaldehyde, and hearts were collected for analyses. In a separate experiment, 8-month-old male WT and MFS mice were perfused with PBS, to use the ascending aorta for mRNA isolation to perform qPCR (*n*  = 3 WT and *n* = 9 MFS). Animal care and experimental procedures were approved by the local independent animal experimental committee for Animal Welfare (permit 97-215-DBC102962) according to the guidelines of the Amsterdam UMC and Directive 2010/63/EU of the European Parliament.

### Real-time qPCR

MFS (*n*  = 6) and non-MFS (*n*  = 6; 3× Loeys-Dietz syndrome, 2× TAA, and 1× BAV) patients’ ascending aorta aneurysm tissues were collected during prophylactic aortic surgery. Permission was granted for the use of the anonymized samples by our local Ethical Board (reference: W16_037#16.052).

Human and murine aortic tissues were crushed in liquid nitrogen and dissolved in Trizol to isolate RNA according to the manufacturer’s protocol. Copy-DNA (cDNA) was synthesized using iScript cDNA synthesis kit (BioRad). Quantitative PCR (qPCR) was performed using SensiFAST SYBR No-ROX Kit (Bioline, Antwerp, Belgium) on a LightCycler 480II PCR platform (Roche). Cycle quantification and primer set amplification efficiency were calculated using the LinRegPCR software package (32). Primers were designed to quantify mRNA by qPCR (primer list provided in Supplemental Table 1, see section on supplementary materials given at the end of this article). Target gene expression was normalized for housekeeping gene Rplp0 (P0). High CD45 (inflammation marker) mRNA expression was considered as aorta pathology and screened for complement factor expression.

### Immunohistochemistry

MFS murine hearts (containing the aortic root with the sinus of Valsalva and the sinotubular junction) were embedded in paraffin and cross-sections were prepared (7 µm). After deparaffinization and rehydration, hematoxylin/eosin (H&E) staining was performed. Immunohistochemical stainings were initiated by quenching endogenous peroxidase activity (20 min in 1% H_2_O_2_) and antigen retrieval (boiling 10 min citrate buffer pH 6). Incubation (overnight, 4°C) with polyclonal rabbit-anti-human C3d-antibodies (Dako, A0063; 1:1000 dilution) or monoclonal rat-anti-mouse C3-antibodies (clone 11H9, Hycult Biotech, Uden, the Netherlands; 1:100 dilution, recognizing C3 and its activation products, C3b, iC3b, C3d). For macrophages, monoclonal rat-anti-mouse Mac-3 antibodies (clone M3/84, BD Pharmingen, Erembodegem, Belgium; 1:30 dilution) were used. Subsequently, the sections were washed and incubated with horseradish peroxidase (HRP)-conjugated anti-rabbit-IgG polymer (BrightVision, ImmunoLogic, Duiven, the Netherlands) or biotinylated rabbit-anti-rat antibodies (Dako, E0468, 1:250 dilution) with subsequent streptavidine-HRP (Dako; 1:500 dilution), respectively, and diaminobenzidine tetrachloride as substrate. For macrophages, HRP-conjugated donkey-anti-rat antibodies (Jackson Laboratories) was used. Sections were rinsed, dehydrated, and embedded in Pertex (HistoLab, Askim, Sweden), and microscopic photographs were taken for quantification (Leica Microsystem and Photoshop CS5/6). The sections were not counterstained with hematoxylin to be able to quantify the amount of staining with QWin software (Leica Microsystem). For C3, the amount of staining in the aortic section included the medial plus the endothelial cell layer, while for the Mac3 staining, just the staining in the medial area was measured to indicate influx of macrophages into the vessel wall (all mice per group; ratio of positive-stained area/total aortic tissue area). Measurements of aortic diameters at each site were performed by tracing the luminal perimeter in H&E sections (*n*  = 3 sections per mouse) with QWin software and subsequently calculating the diameters. The medial area (mm^2^) was measured by tracing the internal elastic lamina (IEL) and external elastic lamina (EEL) in H&E sections and subtracting the IEL from the EEL area. A Masson’s Trichrome staining (Sigma-Aldrich) was performed to reveal enhanced collagen deposition (blue) in the aortic media at the level of the sinotubular junction in the MFS mice.

### Blood cell counts and cytometric bead array

Blood samples were collected in EDTA tubes, and cells were measured in a Beckman Coulter counter. Plasma samples were subjected to the Cytometric Bead Array Mouse Inflammation Kit to analyze interleukin-6 (IL-6), IL-12p70, IL-10, MCP-1, IFNγ, and TNFα (BD Biosciences) and used according to the manufacturer’s protocol.

### Statistical analysis

Graphs were made with Graphpad Prism-5 and represent mean + s.e.m. Unpaired Student’s *t*-test (Gaussian distribution) and the Mann–Whitney U test (non-Gaussian distribution) were used. When a certain parameter could not be detected in a group of mice, the one-sample *t*-test was used. A P-value of ≤ 0.05 was considered significant.

## Results

### Potential modifier genes

In a multigenerational family with various members affected by MFS due to a pathogenic *FBN1* variant, we confirmed the presence of the mutation in* FBN1* (FBN1:NM_000138: exon9:c.T937G:p.C313G) (31) in all six individuals that were selected for WGS. In an effort to identify potential underlying genetic modifiers/risk factors for vascular disease, we mined the WGS data from three family members (p2, p3, and p6, Table 1 and Supplemental Table 2; family tree in Supplemental Fig. 1) with a severe aorta phenotype (aortic surgeries/type A and B dissection) and compared them to three family members with a mild vascular phenotype (p1, p4, and p5 near normal aorta diameters). We searched for protein-altering variants that were present only in the severely affected carriers and absent in the mildly affected carriers. We identified 133 protein-altering variants, of which 4 variants were rare (minor allele frequency (MAF) < 0.001) (Table 1). Moreover, one of the variants stood out, as it was present in a candidate gene for aortopathies and is expressed in the human aorta (33). That variant occurred in the complement Component-1 Subcomponent-R (*C1R*) gene (p.Ser152Leu; MAF = 0.23) in its calcium-binding EGF-like domain (Table 1). This serine is not conserved, but part of a unique stretch of amino acids between the highly conserved first and second cysteine in the domain, containing three serines of which the third is replaced by leucine in our patients; CASRSKSGEEDPQPQC. Replacement of a polar (hydrophilic) serine into a non-polar leucine may impact protein surface exposure or solubility. Sanger validation confirmed that the *C1R* alteration was present only in the severely affected carriers (gray in Table 1). *C1R* encodes for a proteolytic subunit of complement C1-complex. Pathogenic variants in this gene cause Ehlers–Danlos syndrome, periodontal-type, characterized by early-onset periodontitis with premature tooth loss, skin fragility, and occasional aortic aneurysms/dissections (18, 34, 35). These WGS data prompted us to assess the influence of the complement system in aortopathy in MFS syndrome.

**Table 1:** Identification of four rare and one aneurysm-associated gene variant in MFS family members with aortopathy. Interesting variants identified in family members with aortopathy (gray), when compared to family members without aortopathy, yet all harboring the same FBN1 variant.

Chr	Gene	Transcript	AA change	dbSNP	gnomAD freq	P1	P2	P3	P4	P5	P6
1	GPR61	NM_031936	p.R442H	rs190128878	0.0000245	Ref	Alt	Alt	Ref	Ref	Alt
4	GYPA	NM_001308190	p.T44I	rs56172553	0.0004	Ref	Alt	Alt	Ref	Ref	Alt
5	TRIO	NM_007118	p.N1050I	rs200954380	0.0002	Ref	Alt	Alt	Ref	Ref	Alt
12	MMP19	NM_002429	p.G484R	rs145965552	0.0011	Ref	Alt	Alt	Ref	Ref	Alt
12	C1R	NM_001733.7	p.S152L	rs1801046	0.2313	Ref	Alt	Alt	Ref	Ref	Alt

AA change, amino acid change; Alt, heterozygous nucleotide alteration; Chr, chromosome; dbSNP, Single Nucleotide Polymorphism Database; gnomAD freq, Genome Aggregation Database frequency; P, patient; Ref, reference nucleotide.

### Complement factor gene expression

**Figure 1:**
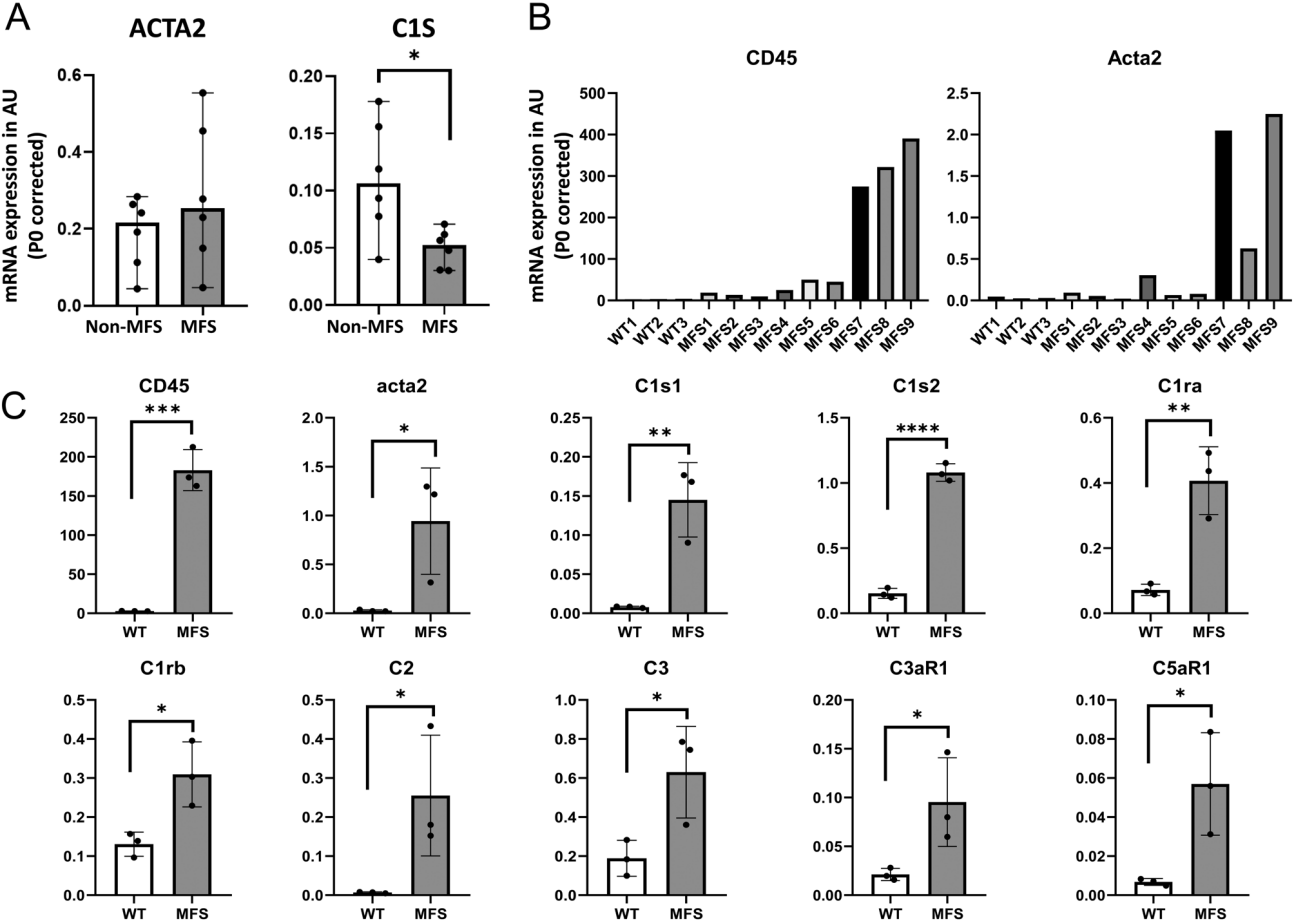
Gene expression in human and murine aortic tissue. A. Smooth muscle cells marker *ACTA2* and *C1S* mRNA is detected in human aortic aneurysm, where *C1S* is less prominently expressed in MFS as compared to non-MFS aneurysm tissue. B. Ascending aortic tissue was utilized of three WT and nine MFS mice to detect mRNA for inflammatory cell marker CD45 to indicate aortopathy in MFS 7–9. In these mice Acta2 mRNA was also enhanced. C. Gene expression was assessed in the three WT mice and in MFS mice seven to nine to study the expression of complement factors, showing that all complement factors examined were enhanced in the MFS mice with aortopathy. Gene expression is expressed in arbitrary units (AU) and corrected for housekeeping gene P0. *P < 0.05; ** *P* < 0.01; ***P < 0.001; ****P < 0.0001.

Human aortic samples were collected from MFS and non-MFS aneurysm patients to study complement factor gene expression *C1R*, *C1S*, *C3AR1*, and *C5AR1* and SMC gene *ACTA2*. ACTA2 mRNA was uniformly expressed in non-MFS aortas but was variable in the MFS aortas (Fig. 1A). Of the complement factor genes examined, only C1S was clearly expressed in MFS, at lower levels when compared to the non-MFS aneurysm samples.

In the *Fbn1* ^C1041G/+^ MFS mice, the aortic root is uniformly enlarged, while dilatation in the ascending aorta is not always observed. However, for practical reasons, we isolated RNA from the ascending aortae of mice; thus, we needed to discriminate between diseased and non-diseased MFS aortas. We isolated aortas of nine MFS mice and three WT mice and used CD45 expression as a readout for aortopathy since it is known that inflammatory cells (CD45+) are present in affected MFS aorta. In 3/9 MFS mice, enhanced CD45 was present and we compared gene expression of these 3 mice for Acta2 and complement factors, to the WT aortae (*n*  = 3). Interestingly, the mice with high CD45 also showed enhanced Acta2 expression (Fig. 1B). All complement genes tested, namely *C1s1*, *C1s2*, *C1ra*, *C1rb*, *C2*, *C3*, and their receptors *C3ar1* and *C5ar1*, were significantly enhanced in the CD45+ MFS aortas, suggesting a potential role in aortopathy (Fig. 1C). In mice, gene duplication resulted in two forms of C1s and C1r, of which C1s2 is most significantly increased in diseased MFS aorta. Expression levels of complement factors in individual mice (Supplemental Fig. 2) show that most factors were already expressed in MFS mice 5 and 6 with only slightly increased CD45 expression, thus preceding an enhanced influx of inflammatory cells.

### Complement deposition and activation

**Figure 2:**
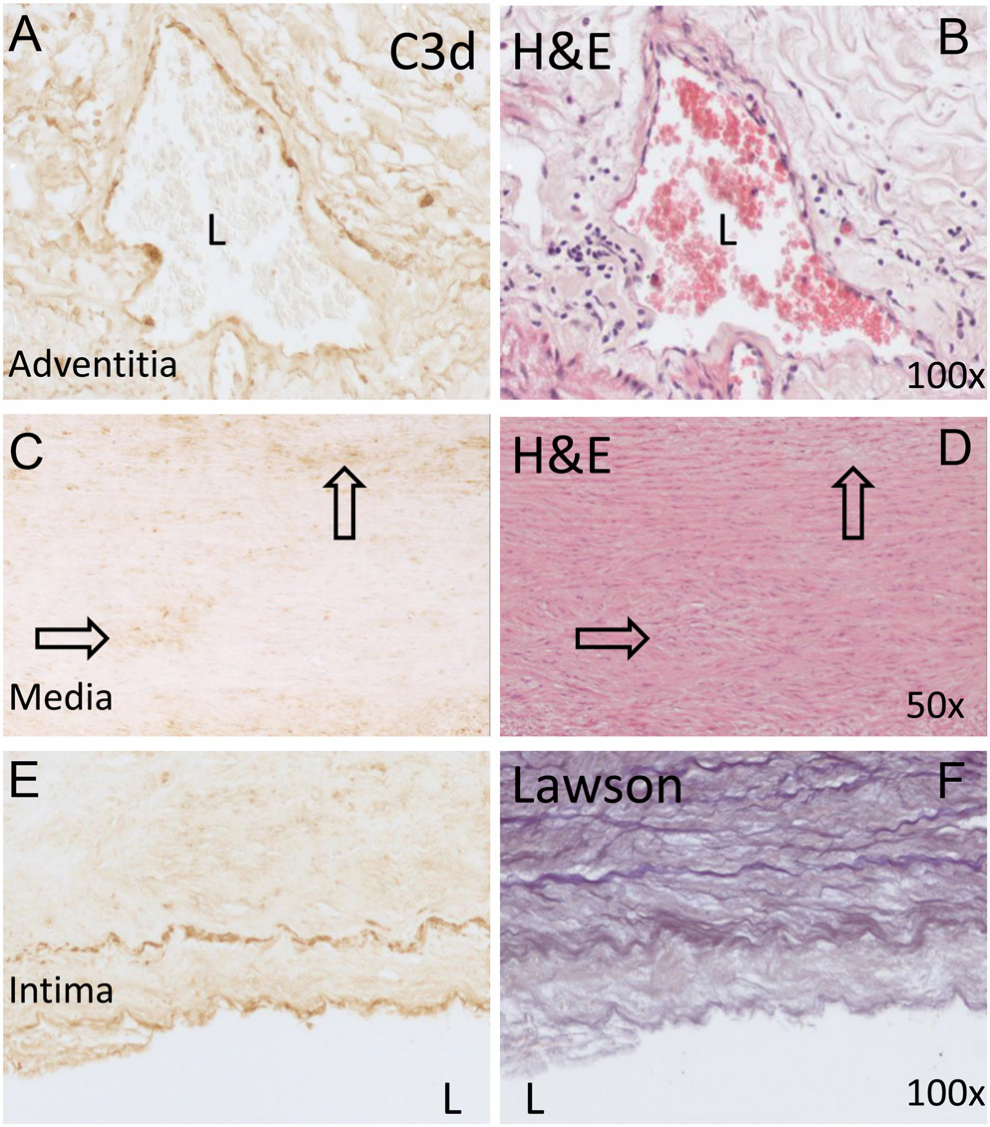
Complement activation in human MFS patient material. A. C3d-stained tissue section, showing the adventitia from the ascending aorta of an MFS patient with a cysteine mutation in the *FBN1* gene, where positive C3d staining (brown) of inflammatory and endothelial cells is observed in and around the vasa vasorum. B. Hematoxylin and eosin (H&E)-stained section (corresponding to A). C. C3d staining in the aortic media, showing positive C3d staining in areas of SMC damage (arrows indicate stained areas). D. H&E-stained section of the media (corresponding to C). E. C3d staining in the intima of an MFS patient is depicted, showing that the internal elastic lamina and the endothelial cells are positive for C3d. F. Lawson-stained intima, showing the elastic lamellae (corresponding to E). L, lumen; magnification 50× or 100×.

In human MFS aorta, C3d-immunostaining was performed as an indicator for complement activation (Fig. 2A, C and E). Complement activation is observed in the adventitia, media, and intima (brown). In the adventitia, C3d co-localized with endothelial cells in the vasa vasorum and occasional inflammatory cells surrounding it (Fig. 2A and B). The media showed C3d in areas of SMC-loss (Fig. 2C and D; arrows). In the intima, C3d is present at the internal elastic lamina and the endothelial cell lining (Fig. 2E and F). The binding of complement factors to elastic fibers has been shown previously in mice (36). The internal elastic lamina is thicker as compared to the medial elastic lamellae (37). This difference in structure may explain why C3d co-localized only with the internal elastic lamina.

**Figure 3:**
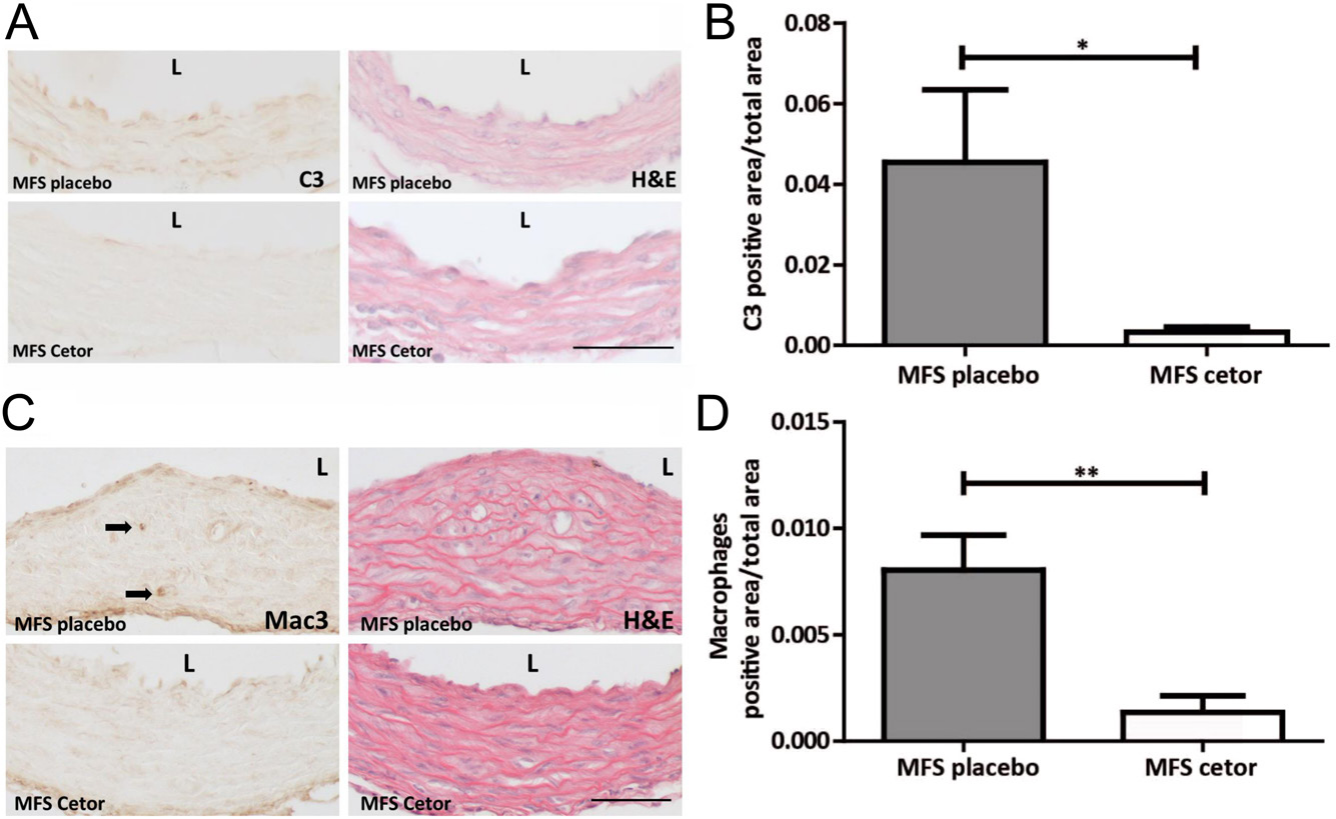
C3 and macrophage staining is less abundant in Cetor®-treated MFS mice. A. The sinus of Valsalva of an MFS placebo mouse is stained for C3 and shows positive endothelial cells and C3 deposition in the media, which is almost absent in the Cetor®-treated MFS mice (scale bar: 100 µm). For orientation purposes, an H&E-stained consecutive tissue section is shown. B. Quantification of C3 positive area divided by total aortic area of MFS placebo and Cetor®-treated mice, indicating less C3 deposition in MFS mice treated with Cetor®. C. Illustrative photographs of a placebo and Cetor®-treated MFS mouse sinotubular junction sections, stained for macrophages (Mac3, arrows) show reduced macrophage content in the latter (scale bar: 100 µm). D. Macrophage influx into the aortic media is inhibited in the Cetor®-treated MFS mice. (mean ± s.e.m. of *n* = 8 mice per group).

To assess whether complement inhibition affects aortic dilatation in MFS mice, we administered C1-INH Cetor® weekly during 2 months, starting at 2 months old. This weekly admission strategy was performed previously in a murine restenosis model, where vascular disease was inhibited (24). C3 staining was performed on aortic sections in the valve areas and revealed the presence of C3 in SMCs in the media and in endothelial cells at the luminal surface (Fig. 3A). Quantification of positive-stained area divided by the total area resulted in diminished C3 deposition in the Cetor®-treated mice (Fig. 3A and B; *P* = 0.038). Macrophage influx into the aortic wall was more abundant in MFS mice, as observed previously (38). Cetor® treatment decreased the macrophage content in MFS aortae (Fig. 3C and D; *P* = 0.003).

### Circulating TNFα is reduced by Cetor®

The effect of Cetor®-treatment on various inflammatory markers in plasma revealed that TNFα was undetectable in Cetor®-treated mice and was significantly reduced when compared to WT or MFS placebo mice (Supplemental Fig. 3A; *P* = 0.028 and *P* = 0.047, respectively). The mice did not show a difference in body weight, while the number of white blood cells was increased in MFS mice due to enhanced lymphocytes, yet this remained similar after Cetor® treatment (3B,C). This difference in lymphocytes between WT and MFS may be explained by enhanced (local) transforming growth factor beta (TGFβ) levels in MFS patients and mice (39, 40) since TGFβ is involved in immune cell development and activation (41).

### C1-INH Cetor® does not affect aortic dilatation

**Figure 4:**
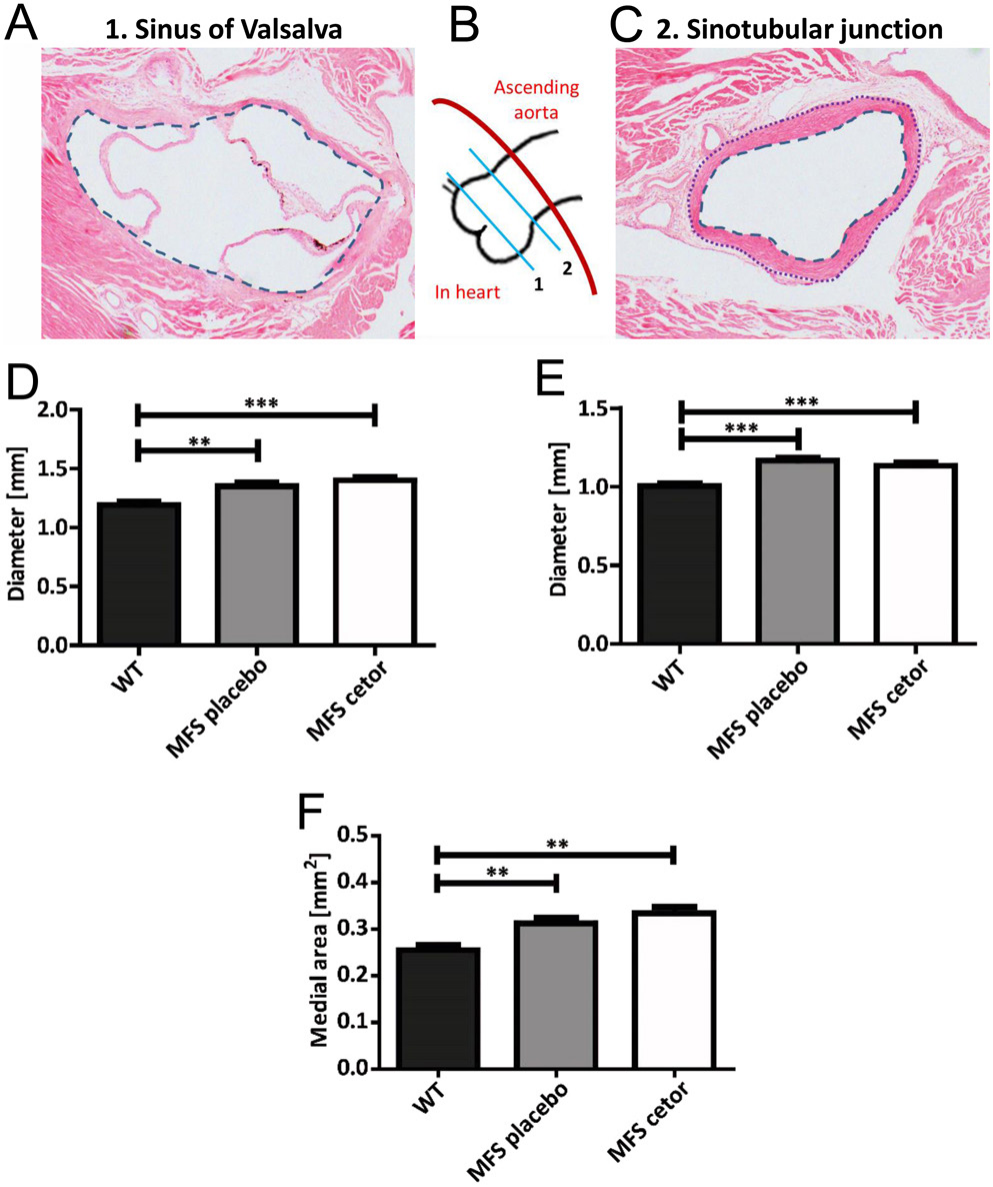
Aortic diameter and medial thickening are not influenced by Cetor® treatment. A. An example of a sinus of Valsalva section, with an indication of luminal perimeter tracing in the blue dotted line to calculate the diameter (H&E) (corresponding to cross-section line 1 in the schematic drawing in B). B. Schematic drawing of the aortic root and ascending aorta indicating the sinus of Valsalva (1) and the sinotubular junction (2). C. An example of H&E staining of the sinotubular junction (corresponding to cross-section line 2 in the schematic drawing in B), with an indication of luminal perimeter tracing in the blue dotted line to calculate the diameter. Subtraction of the area within the blue dotted line from the area within the purple dotted line provides the medial area to determine medial thickening. D. Aortic diameter (mm) at the level of the sinus of Valsalva and E. sinotubular junction in WT, MFS placebo, and Cetor®-treated MFS mice is not reduced by Cetor®. F. Medial thickening (area in mm^2^) in the aortic root is not diminished in Cetor®-treated MFS mice. (*n*  = 8 mice per group)

Aortic dilatation is the most important vascular parameter in MFS. Luminal circumference was measured on H&E-stained sections, from which the diameter is calculated (7) at the level of the sinus of Valsalva (Fig. 4A and B (1)) and sinotubular junction (Fig. 4B and C (2)). The aorta was significantly larger in MFS placebo mice when compared to WT mice at both locations (Fig. 4D and E; *P* = 0.004 and *P* < 0.001, respectively). Two-month treatment with C1-INH Cetor® did not affect these diameters in MFS mice (Fig. 4D and E; both *P* < 0.001). To study whether medial thickening was influenced by Cetor®, the total medial area was measured. In MFS placebo mice, medial thickening was significantly increased when compared to WT mice but did not change upon Cetor® treatment (Fig. 4F; *P* = 0.006 and *P* = 0.001). Medial thickening was in part caused by enhanced collagen deposition in the aortic media in MFS mice, irrespective of Cetor treatment (blue staining in Supplemental Fig. 4).

## Discussion

In this study, we detected an interesting variant of unknown significance for *C1R* by WGS in an MFS family that segregated with severely affected family members. Since *C1R* is an aneurysm-associated gene, we hypothesized that complement activation could enhance aortopathy in MFS. We, therefore, studied mRNA expression of complement factors in human and murine aortic tissues, where C1S was detected as most significant in both. Moreover, on the protein level, C3 was demonstrated in MFS aortic tissues, as a marker for complement activation. Treatment of MFS mice with the C1-INH Cetor® diminished aortic complement deposition, macrophage accumulation, and serum TNFα; however, aortic dilatation and medial thickening were not attenuated. Hence, there seems no prominent causal role for complement activation in aortic aneurysm progression in *Fbn1* ^C1041G/+^ mice.

During aneurysm formation, replenishment of lost aortic SMCs is necessary for aortic healing and is accomplished by promoting SMC proliferation. Yet, we previously demonstrated an inhibitory effect of C1-INH Cetor® on SMC proliferation in a murine vein-graft stenosis model (24), which may explain why Cetor® did not prevent aneurysm formation.

Cetor® treatment resulted in less inflammation, as observed by reduced circulating TNFα and less aortic macrophage accumulation. TNFα has a crucial function in chronic inflammatory diseases such as rheumatoid arthritis, inflammatory bowel disease, and atherosclerosis (42). However, inhibition of this signaling pathway in the angiotensin II-induced AAA mouse model showed a trend toward enhanced incidence of aortic dissection (43). It was suggested that a moderate pro-inflammatory stimulus may be necessary to promote vascular repair (43). In murine AAA models, where excessive aortic inflammation is the driver of disease, blocking complement activation resulted in less aneurysm formation (14, 15). However, we demonstrated previously that in MFS mice, which have low levels of inflammation as compared to AAA models, the influx of macrophages into the aorta was inhibited by AT1R blocker losartan, T-cell specific inhibitor abatacept, and corticosteroid methylprednisolone (38). Yet, only losartan reduced aortic dilatation, showing that inhibition of aortic inflammation in MFS mice is not crucial to reduce aortopathy. Together these data support the need for balanced inflammatory cues to optimize aortic repair and inhibit aneurysm growth.

Also, endothelial cells are influenced by C1-INH Cetor® since it preserves endothelial cell function and survival in mice (24, 44). Endothelial dysfunction has been observed in MFS patients, where flow-mediated dilation is negatively correlated to aortic diameter (45). In MFS mice, decreased endothelial nitric oxide synthase (eNOS) activity is observed with consequences for aortic relaxation/contractility (46). While C3-positive endothelial staining was diminished by Cetor® treatment, this did not rescue aortopathy in MFS mice, indicating that in our MFS study with resveratrol, it protected endothelial cell function probably differently (7) than Cetor®, for example, by restoring eNOS (47, 48).

Next to its function as a complement inhibitor, Cetor® blocks proteases whereby renin-mediated angiotensin-II generation is inhibited (8, 25, 26). Since AT1R signaling is involved in aneurysm growth in MFS mice, but we do not observe reduced aneurysm growth with C1-INH Cetor®, it may be concluded that the observed AT1R signaling in MFS mice is not dependent on renin but rather mediated by a local cue such as stretch-induced activation of AT1R (49).

In conclusion, while the complement system has a role in pathological remodeling of the vascular wall (50), we here show the presence of complement cascade components in the MFS aorta and that inhibition of complement activation by Cetor® reduces complement deposition and inflammation. Nevertheless, Cetor® does not reduce aortic medial thickening and aortic aneurysm formation in MFS mice. Thus, these data also suggest that the identified *C1R* variant p.Ser152Leu is likely benign and not involved in the promotion of aortic disease in our MFS family. However, if the detected variant in *C1R* would induce unwanted binding and cleavage of collagen I and III (18), then this variant may still be involved in aortic complications, which is not studied here. Taken together, C1-INH Cetor® is not considered a suitable candidate drug to treat aortopathy in MFS.

We believe that not only positive studies in disease models should be published. The knowledge of pathways that are not causal for disease progression is just as valuable and will also provide scientific insight. Moreover, it will correct publication bias and reduce resources spent and animal lives lost on duplication of studies.

## Supplementary Material

Supplementary Material

## Declaration of interest

The authors declare that there is no conflict of interest that could be perceived as prejudicing the impartiality of the research reported.

## Funding

This work was supported by an unrestricted grant from Shire http://dx.doi.org/10.13039/100007343 – ViroPharma Incorporated (RBP http://dx.doi.org/10.13039/100004364/HWN/PAK), AMC Graduate School PhD-Scholarship (SH), China Scholarship Council http://dx.doi.org/10.13039/501100004543 PhD-grant (SL), ZonMw http://dx.doi.org/10.13039/501100001826-MKMD grant-114024058 (VdW), Amsterdam Cardiovascular Sciences Out-of-the-Box grant-2019 (ACH/VdW), Department of Medical Biochemistry (CJMdeV).

## Author contribution statement

SH, VdeW, RBP, HWN, PAK, ACH, MJHB, and AVP were involved in the design of study; SH, SL, MH, DK, AVP, AHGD, and DRK were involved in acquisition and analysis; SH, SL, VdeW, CJMdeV, AVP, and PAK drafted/modified manuscript/figures; CJMdeV (Head Dept. of Medical Biochemistry) financially supported the study.
