# Supplementary material for: No prominent role for complement C1-esterase inhibitor in Marfan syndrome mice
Source: Vasc Biol. 2022 Oct 24;4(1):40–9. doi: 10.1530/VB-22-0016 (PMC9782404; doi:10.1530/VB-22-0016)
Supplement: Supplementary Material [file supplementary_material.pdf]

Supplemental Table 1 with PCR primer sets:

HUMAN	Forward primer (5' - 3')	Reverse primer (5' - 3')
RPLP0 (P0)	TCGACAATGGCAGCATCTAC	ATCCGTCTCCACAGACAAGG
ACTA2	GACAATGGCTCTGGGCTCTGTAA	ATGCCATGTTCTATCGGGTACTT
C1R	GGTCCACCAGCTCTCAAACT	TGGCAAGCACCGAGGAATC
C1S	TGTCAAAGCAATGGAAAGTGGAG	TGCTCTCTGGGTCTTCAACT
C3AR1	TTGTGCTTAGCCTGTTTTGCTC	TGGAGAGAATTACTGGGGGCT
C5AR1	AGCCCAGGAGACCAGAACAT	TTATCCACAGGGGTGTTGAGG
MOUSE		
Rplp0 (p0)	GGACCCGAGAAGACCTCCTT	GCACATCACTCAGAATTTCAATGG
Acta2	TCGGTGGCTCCATCCTGGCT	TGCTAGAGGCAGAGCAGGGGG
CD45	CTGCGAGCCCAGCTCCAACC	ACCACTGCACAGCCATGTTCTTTCA
C1ra	TGAGCTTCAGAAAGATGGGCA	GCGTAGATCCGGTGGATAGG
C1rb	GACCTTCCACACAGACTTCTCC	CACTGAGTTGGGCTGCGATG
C1s1	GAAGGAAGAGGGAAAGACAAGG	GCTTGCTTTGGTAGTGAGGGA
C1s2	TCAGAGAGCTGTGCATACGAC	TATAATGGGGGAGTTGGGGCT
C2	CCTGGGCATGGAGACCTCTG	TTCTGGTGACTGCTTTCTTGGG
C3	GATGACCCAAATGGCCTGGAA	GGACCCCAAAAATCACGAAGG
C3aR1	TGGAGCCTTTGGATTCCATCT	GGAGGCAATGTCTTGGGGTT
C5aR1	CGCTGGTTACCACAGAACCC	AATGCCATCCGCAGGTATGTT

Supplemental Table 2

Chr	Gene	Start	End	Ref	Alt	Transcript	AA change	dbSNP	CADD phred	gnomAD freq	P 1	P 2	P 3	P 4	P 5	P 6
1	**GPR61**	110086969	110086969	G	A	NM_031936	p.R442H	rs190128878	13.52	0.0000245	ref	alt	alt	ref	ref	alt
4	**GYPA**	145040841	145040841	G	A	NM_001308190	p.T44I	rs56172553	8051	0.0004	ref	alt	alt	ref	ref	alt
5	**TRIO**	14369565	14369565	A	T	NM_007118	p.N1050I	rs200954380	24	0.0002	ref	alt	alt	ref	ref	alt
12	**MMP19**	56230897	56230897	C	T	NM_002429	p.G484R	rs145965552	20.3	0.0011	ref	alt	alt	ref	ref	alt
12	**C1R**	7242299	7242299	G	A	NM_001733.7	p.S151L	rs1801046	8174	0.2313	ref	alt	alt	ref	ref	alt

Supplemental Table 2. Identification of 4 rare and 1 aneurysm-associated gene variant in MFS family members with aortopathy. Interesting variants identified in family members with aortopathy (gray), when compared to family members without aortopathy, yet all harboring the same FBN1 variant. Chr is chromosome; Ref is reference nucleotide; Alt is heterozygous nucleotide alteration; AA change is amino acid change; dbSNP is Single Nucleotide Polymorphism Database; CADD is Combined Annotation Dependent Depletion; gnomAD freq is Genome Aggregation Database frequency; P is patient.

Supplemental Figure 1.

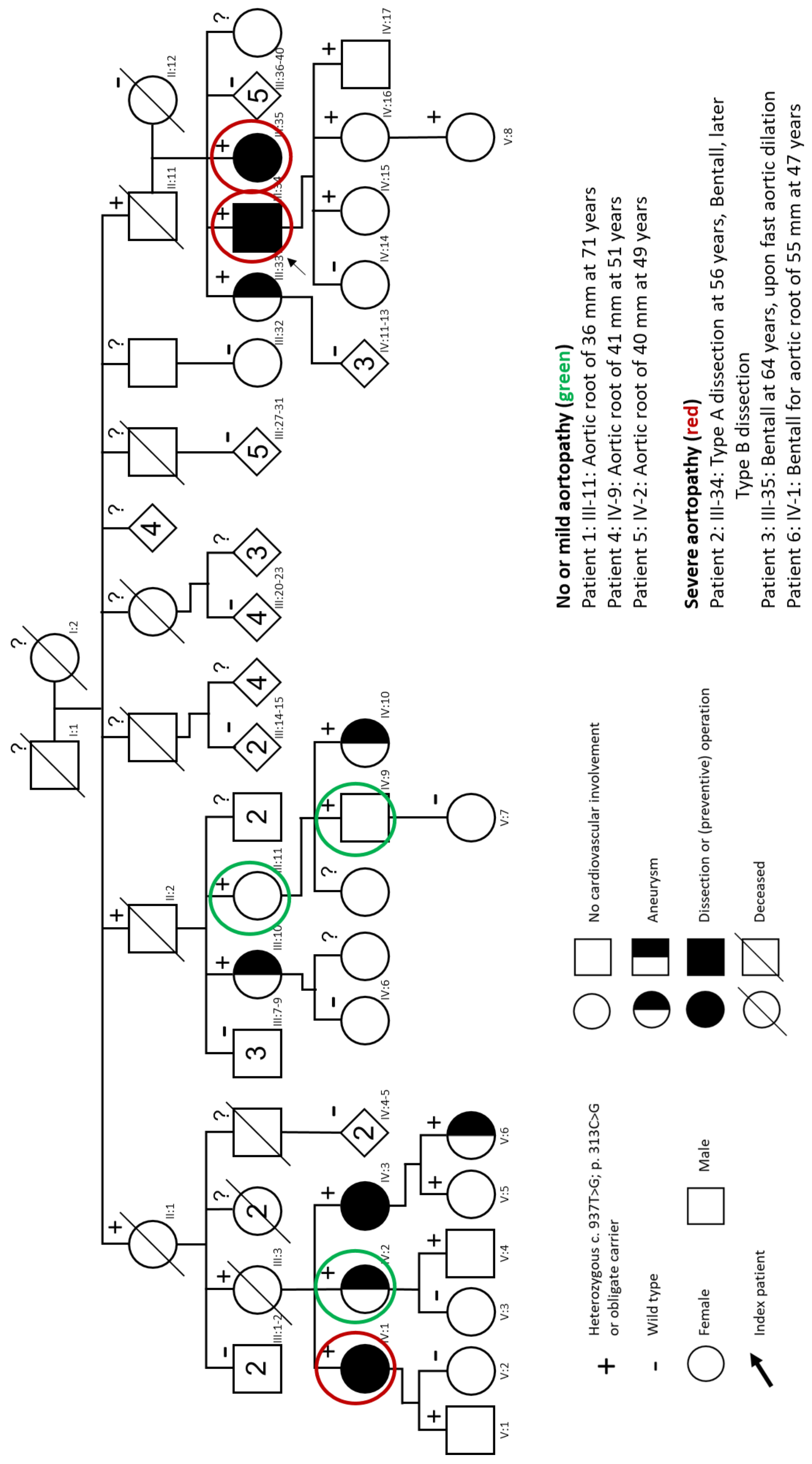

Supplemental Figure 2.

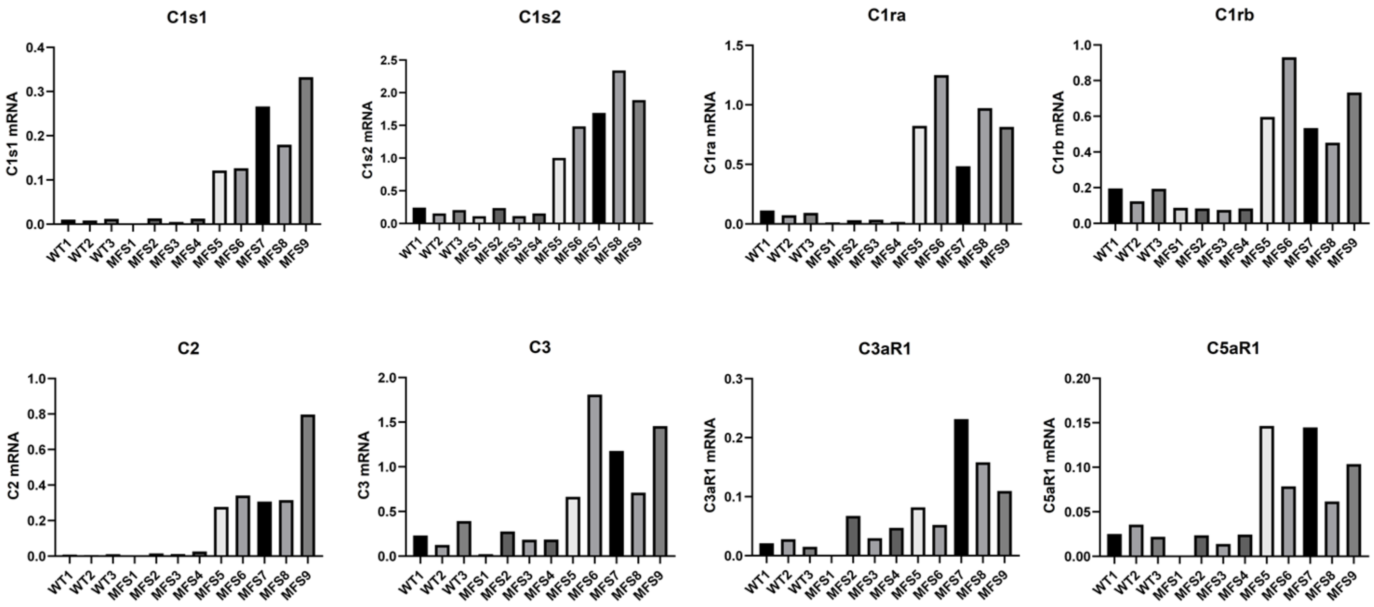

Supplemental Figure 2. Gene expression in wild type and MFS aortic tissue. Most complement factors were expressed in MFS mice 5-9, where only C3aR1 corresponded with the most severe CD45 expression observed in MFS mice 7-9 in Figure 1. In MFS mice 5 and 6, with slightly elevated CD45 expression (Figure 1), complement factor expression in the aorta is already abundant and thus preceded influx of inflammatory cells. Gene expression is depicted in arbitrary units (AU) and corrected for housekeeping gene P0.

Supplemental Figure 3.

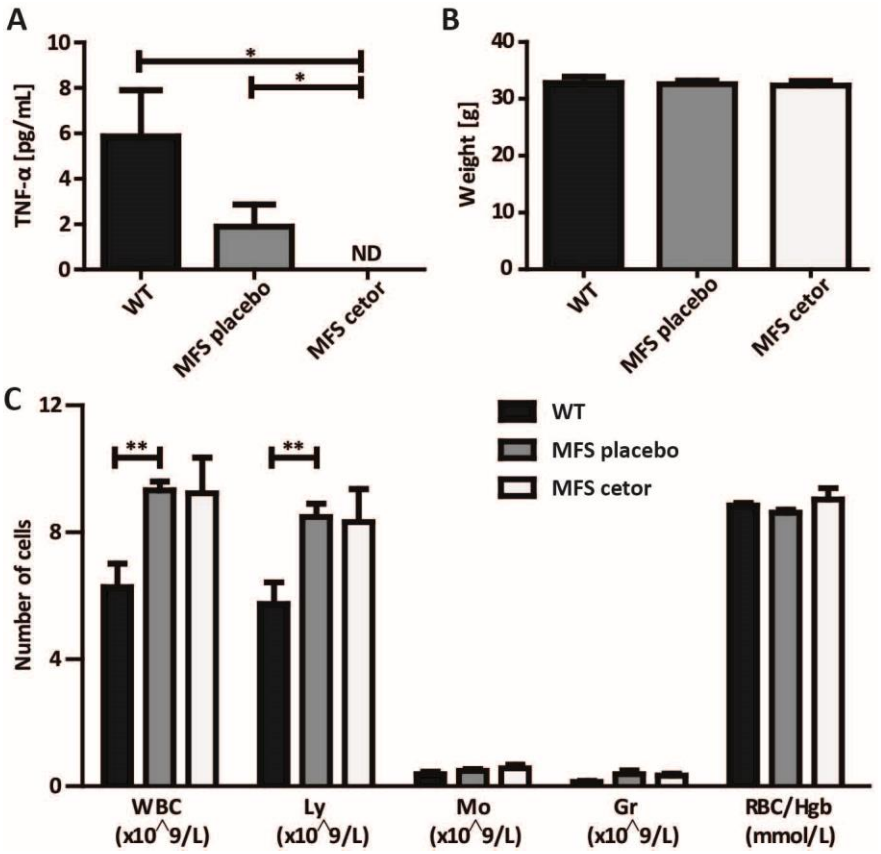

Supplemental Figure 3. Circulating TNFα is reduced in Cetor®-treated MFS mice; weight is not different, neither are the blood cell counts. A. Circulating TNFα is absent in the Cetor®-treated MFS mice when compared to the WT and MFS mice. **B.** Bodyweight (grams) for WT, MFS placebo and Cetor®-treated MFS mice. **C.** White blood cells (WBC) are more abundant in MFS mice when compared to WT mice, due to an increase in blood lymphocytes (Ly). Monocytes (Mo), granulocytes (Gr) and red blood cells (RBC)/Hgb do not show differences. Black bars account for WT mice, grey bars for MFS placebo and white bars for Cetor®-treated MFS mice. (n=8 mice per group) *$p<0.05$; **$p<0.01$

Supplemental Figure 4

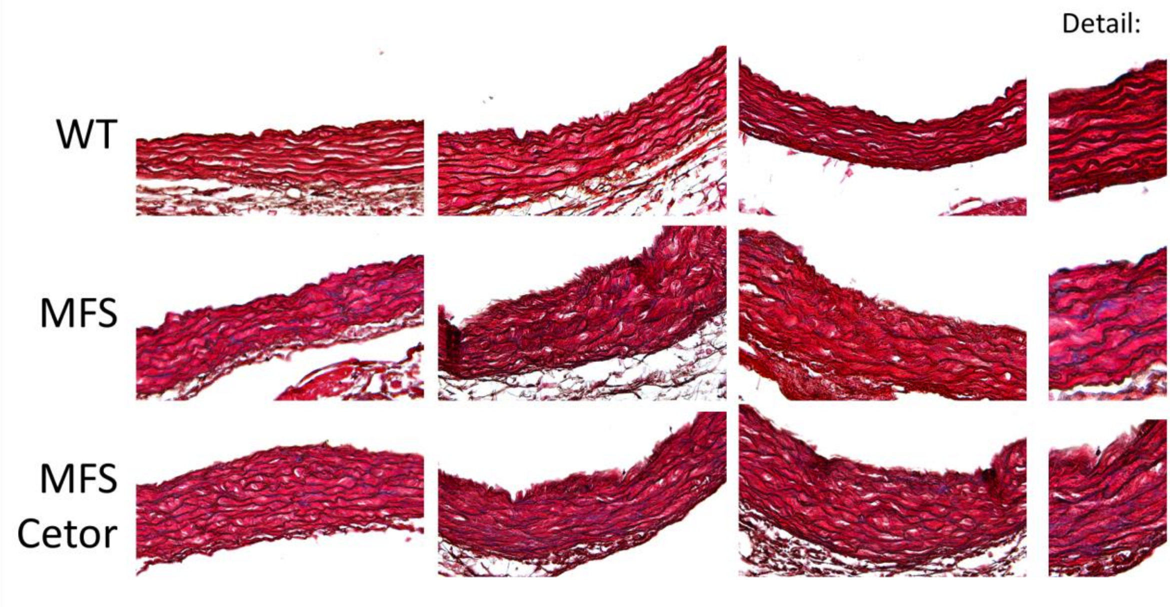

Supplemental Figure 4. MFS mice have enhanced collagen deposition in the aortic media irrespective of Cetor treatment. At the location of the sinotubular junction, the MFS mice reveal occasional blue collagen staining in the aortic media, showing enhanced extracellular matrix deposition, which may in part be responsible for the medial thickening. (examples of n=3 mice per group) Detail reveals a zoom to appreciate the blue collagen staining for each condition.
